# Immunotherapy -2079. Comparison of house dust mite specific IgE and IgG4 between patients receiving rush immunotheraopy and conventional immunotherapy

**DOI:** 10.1186/1939-4551-6-S1-P161

**Published:** 2013-04-23

**Authors:** Ji-Ho Lee, Sang-Ha Kim, Won Yeon Lee, Suk Joong Yong, Kye Chul Shin, Myoung Kyu Lee, Chong Whan Kim

**Affiliations:** 1 Internal Medicine, Yonsei University Wonju College of Medicine, Wonju, South Korea

## Background

When the specific immunotherapy with house dust mite was done in patients who had allergic diseases, change of specific immunoglobulin(Ig)E and IgG4 is well known. But, it is not known whether there is change for specific IgE and IgG4 during initial build-up phase and there is difference for antibody between rush immunotherapy group and conventional immunotherapy group during maintenance phase.

## Methods

25 patients who received immunotherapy with Dermatophagoides pteronyssinus and D. farinae were analyzed retrospectively. 14 patients was treated with rush immunotherapy and 11 patients was treated with conventional immunotherapy. The D. pteronyssinus and D. farinae specific IgE, IgG4 was measured at 6, 12, 24, 36 and 48 months, respectively in both groups. Especially, in rush immunotherapy group, specific IgE and IgG4 was measured in 1, 2, 4 days during initial build-up phase to evaluate early change of antibody.

## Results

All patients had rhinitis and some had asthma (n=16, 64%) or atopic dermatitis (n=7, 28%). There is no difference in clinical characteristics between both groups. In rush immunotherapy group, the D. pteronyssinus and D. farinae specific IgE reached a peak at 6months. In conventional immunotherapy group, D. pteronyssinus and D. farinae specific IgE reached a peak at 12months. Specific IgE in both groups after reaching the peak level gradually decreased. The D. pteronyssinus and D. farinae specific IgG4 in both groups was increased annually but specific IgG4 level was high in rush group than in conventional group. In rush immunotherapy group, there is no significant change in specific IgE and IgG4 during initial build-up phase.

## Conclusions

When the immunotherapy with house dust mite was performed, in rush group the specific IgE reached a faster peak than conventional group (6months vs. 12 months). The specific IgG4 was increased annually and was higher in rush group during maintenance phase. In rush group, specific IgE and IgG4 appeared no significant change during initial build-up phase.

